# Use of Machine Learning to Detect Wildlife Product Promotion and Sales on Twitter

**DOI:** 10.3389/fdata.2019.00028

**Published:** 2019-08-27

**Authors:** Qing Xu, Jiawei Li, Mingxiang Cai, Tim K. Mackey

**Affiliations:** ^1^Global Health Policy Institute, San Diego, CA, United States; ^2^Department of Healthcare Research and Policy, University of California, San Diego–Extension, San Diego, CA, United States; ^3^Department of Computational Science, Mathematics and Engineering, University of California, San Diego, San Diego, CA, United States; ^4^Department of Computer Science and Engineering, University of California, San Diego, San Diego, CA, United States; ^5^Department of Anesthesiology, University of California San Diego School of Medicine, San Diego, CA, United States; ^6^Division of Infectious Disease and Global Public Health, Department of Medicine, University of California San Diego School of Medicine, San Diego, CA, United States

**Keywords:** wildlife trafficking, wildlife product sales, social media, twitter, machine learning

## Abstract

Social media is an important channel for communication, information dissemination, and social interaction, but also provides opportunities to illicitly sell goods online, including the trade of wildlife products. In this study, we use the Twitter public application programming interface (API) to access Twitter messages in order to detect and classify suspicious wildlife trafficking and sale using an unsupervised machine learning topic model combined with keyword filtering and manual annotation. We choose two prohibited wildlife animals and related products: elephant ivory and pangolin, and collected tweets containing keywords and known code words related to these species. In total, we collected 138,357 tweets filtered for these keywords over a 14-day period and were able to identify 53 tweets from 38 unique users that we suspect promoted the sale of Ivory products, though no pangolin related promoted post were detected in this study. Study results show that machine learning combined with supplement analysis approaches such as those utilized in this study have the potential to detect illegal content without the use of an existing training data set. If developed further, these approaches can help technology companies, conservation groups, and law enforcement officials to expedite the process of identifying illegal online sales and stem supply for the billion-dollar criminal industry of online wildlife trafficking.

## Introduction

Trade in wildlife goods puts endangered species under constant threat (Alagappan, 1989; The Humane Society of the United States, 2002; Rosen and Smith, 2010). The Internet (including websites, e-commerce platforms, the dark web, and social media) acts to globalize this trade and serves as a convenient conduit for illegal distribution, trafficking, marketing, and sales of wildlife products (Lavorgna, 2014; Xiao and Wang, 2015; Xiao et al., 2017).

This digital wildlife marketplace includes the use of popular social media platforms that enable creation, sharing, and promotion of content including the direct-to-consumer sale of wildlife and related products (Kietzmann et al., 2011; Harrison et al., 2016). The popular microblogging site Twitter has been identified as one of these conduits, with wildlife dealers posting their contact information, URLs of their online stores (or links to e-commerce websites), and photos of the wildlife goods they offer, which are sourced illegally and explicitly include species protected by domestic and international laws (Di Minin et al., 2018a,b; Monkman et al., 2018).

There have been some studies that have detected online wildlife trafficking via data mining or machine learning used to classify wildlife goods, including the use of image recognition (Hernandez-Castro and Roberts, 2015; Austen et al., 2018; Di Minin et al., 2018a,b; Monkman et al., 2018; Parham et al., 2018). Based on these recent studies, machine learning approaches appear to need a large set of labeled data.

In response, this article proposes a method to help identify wildlife traffic with unlabeled data. We describe the use of the biterm topic model (BTM) combined with keywords filtering for detection of potential marketing and sale of two wildlife products. We find that BTM can help detect a relatively low volume but highly relevant group of tweets specific to wildlife trafficking for one species over a short period of data collection. We also outline a set of technology and policy challenges and recommendations to better leverage big data, machine learning, and social media surveillance to combat wildlife trafficking online.

## Methods

The study is divided into four phases including: (1) manual search; (2) data collection; (3) data processing; and (4) data analysis. To explore the utility of this methodology, we selected two highly protected species and their related products which have been reported as offered for sale online: ivory–*Loxodonta* spp. (endangered) and pangolins (eight species are in the range from vulnerable to critically endangered status).

### Ethics Approval

Twitter messages are in the public domain and only public tweets filtered by the Twitter public application programming interface (API) were collected. Hence, no ethics approval was required for this study as we relied on publicly available data and did not include any private messages and there was no interactions between users and researchers. Further, the user name, contact information and the URLs in the tweets are not disclosed in this study for the purpose of de-identifying specific user information.

### Manual Search Strategy

The first phase of our study involved conducting manual keyword searches directly on the Twitter platform. The objective of performing manual searches was to: (1) select proper keywords to be used in the data collection phase; and (2) select sub-keywords for keywords used as filters in data processing phase.

In June 2019, we performed manual searches using keywords and known codewords for both species on the search engine on twitter's public website interface. These keywords and codeword focused on the wildlife product “ivory” and the species name “pangolin” and also included seven codewords for ivory and eight codewords for pangolin. Important to note is that among online wildlife trading, codewords are used for charismatic species, with ivory being one of such example (Alfino and Roberts, 2018). Due to concerns about consumers using codewords to search for and source wildlife products, we do not specifically disclose them in the methods of this study, though they are available upon request from study authors. Based on these search queries, we then examined all visible tweets that were posted in the prior year (up to July 1, 2018). All tweets that directly promoted or sold ivory or pangolin related products were recorded. All the codewords that were used in these tweets with the meaning of “Ivory” or “Pangolin” were selected as our keywords for data collection. Note that for validation purposes, the manual search process was performed twice, separately by two human coders. A codeword was excluded only when both coders had a zero count of relevant tweets for that query.

From the suspected tweets found in manual search results, we also extracted words in the text of the tweet that exhibited high frequency and use. We defined these words as sub-keywords and saved them as keywords filters in the data processing phase.

### Data Collection

Based on results in our manual search phase, we selected two distinct groups of associated keywords for ivory and pangolin which were used to filter tweets from the Twitter public API over a 14-day period (June 19, 2019–July 4, 2019) (Makice, 2009; Morstatter et al., 2013). Our keywords include the species related keywords (“ivory” and “pangolin”) and the selected codewords from manual searches. In addition to the text of the tweets, other metadata (e.g., geolocation, time, # of followers/following) for each post were collected.

In total, we collected 138,357 tweets filtered for these keywords, though the vast majority contained “noise” (i.e., tweets unrelated to online sale or trafficking of wildlife products but that contained the filtered keywords) such as tweets containing news articles, information about animal protection/preservation activities, wildlife-related law and policy, and even animal cartoon characters. We removed all stop-words and special characters unrelated to the main message of the tweet that could make it difficult for our machine learning algorithms to distinguish from non-relevant terms, and generated a codebook by renaming the remaining words in the text with unique corresponding indexes (Silva and Ribeiro, 2003).

### Data Processing

For both the ivory and pangolin dataset, we analyzed tweets using BTM which is an unsupervised machine learning topic model that uses Nature Language Processing (NLP) to categorize short forms of text in a given number of groups (topics) by analyzing the correlations between words and topics (Cheng et al., 2014; Sasaki et al., 2014; Rangarajan Sridhar, 2015; Li et al., 2016). We used BTM as a topic clustering method to categorize similar text into related topic clusters.

**Figure 1:**
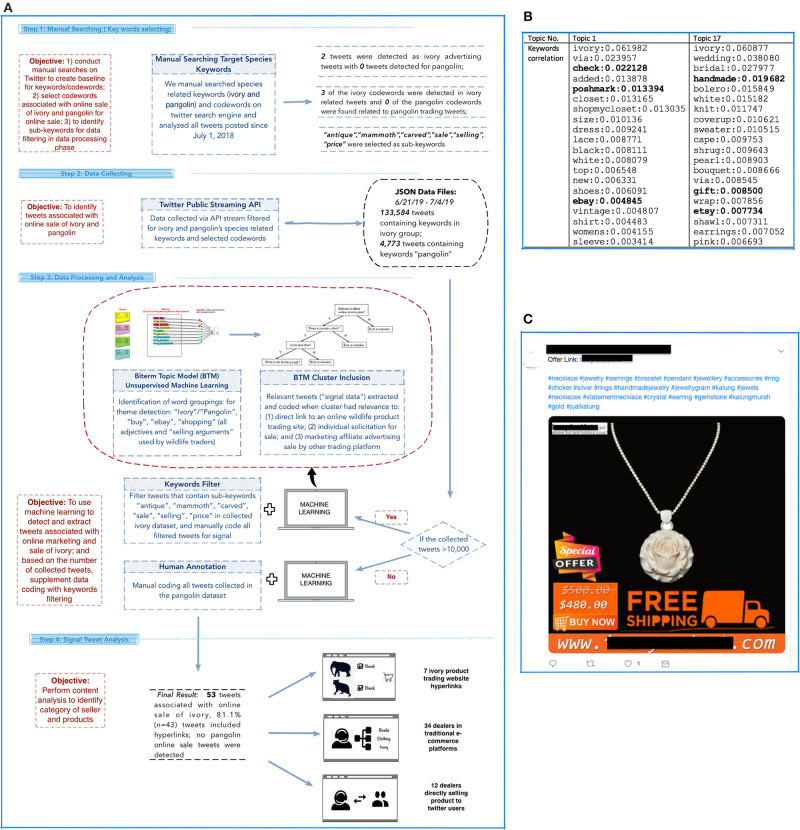
Summary of study methodology. **(A)** Two tweets were detected in manual searches as ivory advertising tweets, no pangolin promote tweets are detected, three codewords were confirmed on twitter that were used for Ivory product; “A total of 133,584 tweets were collected which contained the keywords ‘Ivory' and three selected codewords; 4,733 tweets were collected containing the keyword ‘pangolin.”' BTM was used to detect topics highly corelated with keywords associated with sale and trafficking resulting in two topics possibly associated with ivory dealers who market or sell ivory via Twitter. Base on the size of the dataset, we conducted supplementary analysis (e.g., keywords filtering and human annotation) to detect true positive signals. **(B)** BTM output where we identified two topics with a total of 91 tweets with high correlation with signal keywords. Tweets were then classified using human coding. **(C)** One example of signal after we isolated messages (tweets) associated with sale and trafficking of ivory.

We split all text into a bag of words and then produced a discrete probability distribution for all words for each theme. This distribution places a large weight on the words that are most representative of that theme (Kalyanam et al., 2017). We output the top “*n*” words that had the highest correlation value to represent the topic of the cluster. According to our topic groupings, we identified messages with characteristics we believed were related to online wildlife trafficking (“signal” data) including:

- any word that relates to online trading or potential sale (e.g., buy, sale, shipping, etc.);
- are associated with an online/e-commerce trading platform (e.g., ebay, Amazon, etc.);

We analyzed these two groups of collected tweets separately (i.e., data for “ivory”-related keywords were analyzed separately from “pangolin”-related keywords) in separate BTM processes. In each group of analysis, we tried to isolate, summarize, and further characterize signals from these collected tweets (see Figure 1 for a summary of the methodology).

We conducted BTM analysis by initially setting “*k*” number of topics and “*n*” words per topic. In order to find an appropriate value of “*k*”, we used the coherence score, described below (Mimno et al., 2011). From the total number of clusters generated by BTM, we sought to find highly distinguishable clusters in order to make it easier for a human coder to identify the theme of each topic. First, we use the formula (1) to calculate the correlation between these words, where score(V_i_, V_j_) represents the correlation between two words V_i_ and V_j_, D(V_i_, V_j_) represents the number of texts that contain these two words, D(V_i_) is the number of the text that contain V_i_.

(1) $$\begin{array}{r} \text{score}\left({\text{V}}_{\text{i}},{\text{V}}_{\text{j}}\right)=\text{log}\left(\left(\text{D}\left({\text{V}}_{\text{i}},{\text{V}}_{\text{j}}\right)+1\right)/\text{D}\left({\text{V}}_{\text{j}}\right)\right) \end{array}$$

Both V_i_ and V_j_ belong to the top n words in each topic T, we then calculate the coherence score for each topic using formula (2). Finally, we use the formula (3) to calculate the average of the coherence score for this *k* value.

(2) $$\begin{array}{rcl} \text{coherence}\left(\text{T}\right) & = & \underset{\left(V_{i},V_{j}\right)\;\in \text{V}}{\sum }score\left(V_{i}{,V}_{j}\right) \end{array}$$

(3) $$\begin{array}{rcl} \text{K}-\text{coherence}\left(\text{k}\right) & = & \frac{1}{k}\overset{k}{\underset{m\;=\;1}{\sum }}coherence\left(T_{m}\right) \end{array}$$

V represent the top n words, k represents the number of topics, *T*_*m*_ is the m^th^ topics, K-coherences(k) is the average coherence score for the *k* value.

The coherence score evaluates the similarity between each pair of words in the topics. In order to make the topic more identical, two words with high correlation are expected to be assigned to the same cluster. Therefor the larger the coherence score, the better the topics are extracted. In this study, we test *k* = 5, 10, 15, 20, 25, and 30.

Based on the size of each dataset in a topic cluster with potential signal data, we then combined BTM results with supplemental analysis. If the size of extracted tweets exceeded 10,000 messages, we combined keyword filtering with BTM. We used the same dataset collected from the Twitter API and filtered all tweets that contained the sub-keywords selected from our manual searches. Then we looked for true positive signal from the filtered dataset by manually annotating messages. The signal tweets from keywords filtering and BTM were combined and the duplicated tweets were removed. When the number of the collected tweets was smaller than 10,000 we used BTM and supplemented with human annotation.

We hand coded all tweets and examined messages for illegal wildlife trafficking promoting tweets and combined the results. The criteria for detecting signal in the dataset was determined by assessing if a tweet was posted by a Twitter user and included an offer of sale of a wildlife product(s). For our ivory dataset, we analyzed the text and also visually inspected images of products (if available) in an attempt to determine if they were ivory product sales. In the dataset, either the posted product had a material description noting that the product was made of ivory or the product image was inspected and showed clear Schreger lines that we could identify as probable authentic ivory. For pangolin dataset, we analyzed the text of the product description only. Importantly, though we visually inspected images of products in an attempt to determine if they were authentic ivory product, we were unable to confirm the authenticity of most of the images by inspecting for Schreger lines due to variability of images and types of products. We discarded posts that contained images which were clearly not elephant ivory (i.e., were plastic or were clearly jewelry not made of elephant ivory). Second and third author coded posts independently and achieved a high intercoder reliability for results (kappa = 0.98). For inconsistent results, both authors reviewed the posts together and met and conferred on the correct classification of the post.

## Results

### Manual Search Strategy

After conducting manual searches for ivory, pangolin and the 15 related codewords, we confirmed that two tweets were directly promoting the sale of ivory or suspected ivory products and no twitter messages were detected related to the sale of Pangolin. We excluded four of ivory's and eight of pangolin's codewords due to lack of finding wildlife trafficking related messages. According to the analysis of ivory signal posts detected in our manual search, “antique,” “mammoth,” “carved,” “sale,” “selling,” and “price” were selected as sub-keywords for keywords filtering in data processing.

### Ivory Dataset

**Figure 2:**
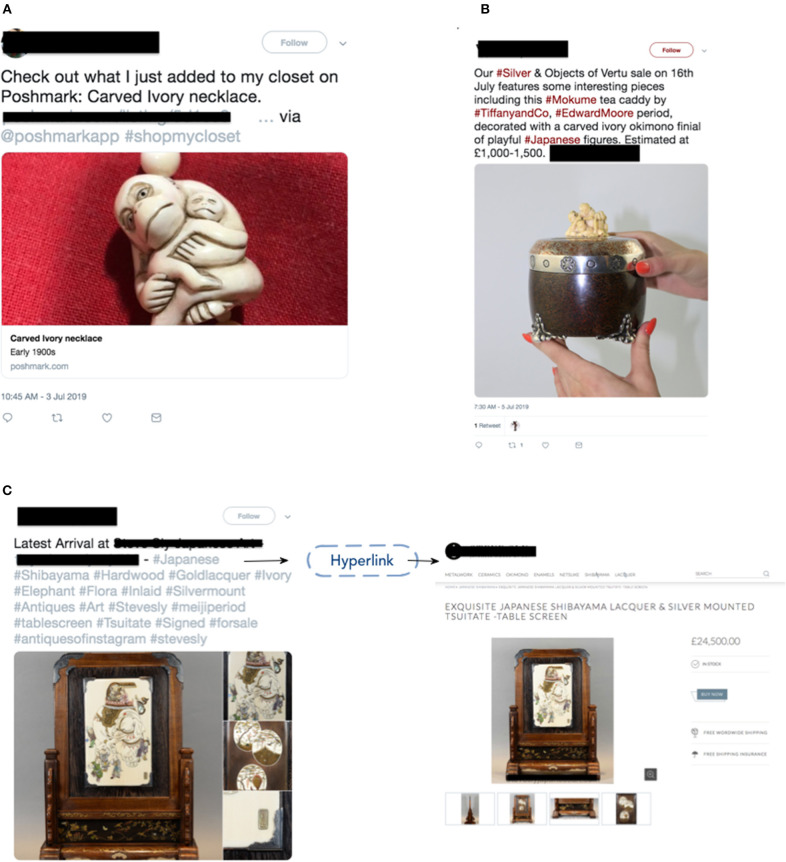
Examples of tweets and websites detected selling ivory online. **(A)** An example tweet selling carved ivory necklace; **(B)** An example tweet selling a tea caddy decorated with carved ivory; **(C)** An example tweet providing hyperlinks that redirected Twitter users to a trading site.

For ivory-related keywords, we collected 133,584 tweets for analysis. Based on the coherence score, when *k* = 25 we retrieved the highest value indicating that topic clusters were distinct. From these clusters we retrieved two topics that appeared to contain signal. The 1,704 tweets in these two topic groupings appeared to be highly associated with marketing, trafficking, and sale of ivory products based on their word groupings. These tweets were then extracted and examined via manual annotation. After manually annotating the 1,704 tweets, we confirmed that 43 tweets had true positive signals (see Figure 2 for examples).

Due to the large volume of tweets in the ivory dataset (138,357 ≥ 10,000), we also supplemented our BTM analysis with keywords filtering. Based on filtering for sub-keywords, 3,289 tweets were identified that contained at least one of the keywords of “antique,” “mammoth,” “carved,” “sale,” “selling,” and “price.” After manual annotation, 46 tweets were confirmed as true positive signals.

Based on the combination of these two approaches (BTM and manual annotation and sub-keyword filtering and manual annotation) a total of 53 tweets were confirmed that directly promoted or sold ivory/suspected ivory products. Eleven of these tweets were confirmed by visual inspection and detection of clear Schreger lines as authentic ivory products. The remaining 42 tweets were classified as ivory sales by the product description and/or its associated image that appeared probable as an elephant ivory product based on its general product type and text description but could not be verified as authentic ivory. Forty-three of these tweets provided hyperlinks that redirected Twitter users to a trading/e-commerce site or other social media platform (twenty-three Etsy, five Poshmarkapp, six eBay, two Instagram, five IvoryAndArt—an ivory art store, one Woolley &Walls- an auctioneer website, and one Krafty Max Originals—a handcrafted jewelry store). These external sites provided access to more detailed information about purported ivory products; and two of these URLs redirected potential customers to an external hosted website that includes other purported ivory products (both of these websites included an online shopping cart for ordering).

We also analyzed all false positive messages, which though included signal keywords and were highly correlated to signal, were not related to online marketing or sale. Some examples of false positives include:

- News related: e.g., “Illegal ivory found on sale in 10 European countries–The Guardian,” (a news article reporting the discovery of illegal ivory sales in Europe);
- Wildlife conservation: e.g., “Never buy ivory. No one needs ivory except its original owners,” (an animal conservation post);
- Ivory color: e.g., “Pearl Necklace, Multi Strand Weddings,” (an advertisement for necklace jewelry that has ivory color);
- Geographic name: “A cocoa price floor of $2,600 per ton agreed by Ivory Coast and Ghana,” (a news related to Ivory Coast cocoa price);
- Museum art introduction: “ivory-carved salt cellar fifteenth century benin–British museum,” (an art introduction from museum).

### Pangolin Dataset

Though we did not find wildlife trafficking messages in manual searches, we wanted to collect and analyze the term “pangolin” for potential trafficking in a larger dataset. However, similar to our manual search results, we did not detect signal tweets associated with online marketing or sale for this species keyword. In total, we collected 4,773 tweets which contained “pangolin.” After the process of BTM, we were not able to detect signal keywords in our output of 10 topic groups. Considering the small size of the dataset (4,773 ≤ 10,000), we supplemented the analysis with manual annotation of the entire corpus and did not detect any true signal.

Similar to our ivory results, we found a large amount of false positive tweets regarding news including the establishment of a National park in Malaysia that could be a pangolin sanctuary and the newly released pangolin population status updated by the World Wildlife Fund; documentaries about pangolin's life and people working on protecting pangolin; and tweets related to pangolin cartoon characters. However, the largest amount of tweets were related to the recognition of pangolin (pangolin's pictures or videos were posted within the tweet, while the text asked the name of the species).

## Conclusion

Detecting online wildlife trafficking is a race against time with criminal actors. Specifically, online traders can self-delete information contained in social media posts after a sale or trade has been completed. In response, many wildlife conservation organizations and tech-companies are reportedly taking action to combat wildlife trafficking online, with The Coalition to End Wildlife Trafficking Online representing one such example.

Importantly, machine learning approaches to quickly detect sales, and immediate action by platforms in conjunction with digital surveillance and in partnership with law enforcement should be further developed. This study provides a potential approach to part of this challenge, using an unsupervised machine learning topic model, which could be used as part of a broader surveillance and monitoring strategy to detect and isolate suspected wildlife trading from large volumes of data.

To improve on these approaches and enable scale-up of technology-based solutions, one of the key challenges that needs to be addressed is lack of harmonization of international policy and national legislation around conservation and wildlife trafficking. Detecting online wildlife trading is just the first process in combating this illicit trade. Critically, further analysis is required to determine if a sale or offer for sale is in fact illegal, particularly if applicable legislation varies based on the jurisdiction of the seller or type of species. For example, the sale of ivory products are prohibited in almost all countries. However, Japan is a notable exception. Additionally, even under the Convention on International Trade in Endangered Species of Wild Fauna and Flora (CITES, 2017), a multilateral treaty that entered into force on July 1, 1975, and currently (July 2, 2019) enjoys membership from 183 state parties^1^, actual implementation of treaty obligations varies (Hastie and McCrea-Steele, 2014).

Despite the potential benefits of technology such as machine learning, combating the global criminal trade in wildlife trafficking will require multi-stakeholder collaboration and coordination. This is an explicit objective of the Global Coalition to End Wildlife Trafficking Online, which held a September 2018 meeting that brought various stakeholders from conservation groups, technology companies, and academic researchers together to help bring an end to this trade. Preliminary results and intelligence garnered from our machine learning approach can help save time and effort for detecting illegal sales on social media, but also requires additional manual work to verify criminal activity, interdict these activities in the field and in the e-commerce marketplace, and also requires public outreach and education to address the consumer demand side of the problem.

However, in the simplest terms, no endangered species trafficking or sale should ever be allowed via online channels, a challenge we hope this study can begin to address for the sake of current and future global wildlife conservation efforts.

## Limitations

This study has certain limitations. First, we only collected data over a short time period in order to assess the feasibility of the methodology. This resulted in a relatively small volume of tweets being identified as signals. Future studies should collect larger volumes of data over a longer period of time. Our study was limited to English language tweets, though other languages may be used by illegal wildlife traders. Additionally, the number of known codewords for illegal wildlife products are limited. Future studies should analyze multi-language text and slang terms if possible and also employ combination of text classification and image recognition (including the use of multi-modal approaches) to improve accuracy of signal detection. Further, we were unable to verify the authenticity of ivory products offered for sale online through further inspection, though we attempted to visually inspect for Schreger lines. Future studies should develop additional detection and verification methodologies based on specific product features that can be extrapolated from different types of images.

## Data Availability

Data was collected from the Twitter public streaming API per the terms of use and is available upon request from authors subject to limitations of sharing per Twitter's terms of use.

## Author Contributions

This manuscript has been seen by all authors, who have approved its content. This piece is not under consideration in any other forum. We note that with respect to author contributions, QX, JL, MC, and TM jointly designed the study, collected data, and worked on the manuscript text. All authors contributed interpretation of the study findings.

### Conflict of Interest Statement

The authors declare that the research was conducted in the absence of any commercial or financial relationships that could be construed as a potential conflict of interest.

## Footnotes

^1^“The number of Parties to CITES may fluctuate because of geopolitical changes such as the unification of two Parties, or the division of a State into two or more separate States”, see https://www.cites.org/eng/disc/parties/index.php.
